# Vitamin D deficiency among patients with pulmonary hypertension

**DOI:** 10.1186/s12890-019-1011-7

**Published:** 2019-12-21

**Authors:** Andrés N. Atamañuk, Diego F. Litewka, Sergio J. Baratta, Ignacio M. Seropian, Graciela Perez Prados, Miguel O. Payaslian, Juan P. Ortiz Fragola, Pilar Escribano Subias

**Affiliations:** 1 División Cardiología y Servicio de Neumonología, Hospital General de Agudos “Juan A. Fernández”, Ciudad Autónoma de Buenos Aires, Argentina; 2 Instituto de Cardiología y Terapéutica Cardiovascular, Hospital Universitario Austral, Buenos Aires, Argentina; 3 Servicio de Hemodinamia, Hospital Italiano de Buenos Aires, Buenos Aires, Argentina; 4 Servicio de Cardiología, Hospital Universitario 12 de Octubre, Madrid, Spain

**Keywords:** Pulmonary hypertension, Pulmonary arterial hypertension, Vitamin D, Vitamin deficiency

## Abstract

**Background:**

There is little information about vitamin D (Vit D) deficiency in patients with pulmonary hypertension (PH). The objective of this study was: 1) compare Vit D levels between patients with PH, left ventricular failure (LVF) and healthy subjects (HS); 2) correlate, in patients with PH, Vit D levels with prognosis-related variables, such as the 6-min walk test (6MWT).

**Methods:**

Vitamin D levels were measured in a cross-sectional study in 126 patients from one of three groups: patients with PH (*n* = 53), patients with LVF (*n* = 42) and healthy subjects (*n* = 31). In all groups, 8-h fasting blood samples were obtained in the morning. In the PH and the LVF group, functional class (WHO criteria), metres covered in the 6MWT and echocardiographic parameters were analysed. In the PH group, plasma N terminal pro B type natriuretic peptide (NT-proBNP) level was analysed and a complete haemodynamic evaluation by right heart catheterisation was made.

**Results:**

Mean Vit D levels were lower in PH than in both other groups (ng/ml, mean ± SD): PH 19.25 ± 10, LVF 25.68 ± 12, HS 28.8 ± 12 (PH vs LVF *p* = 0.017, PH vs HS *p* = 0.001 and HS vs LVF *p* = 0.46). Vit D deficiency prevalence was higher in PH as compared to the other groups (PH 53.8%, LVF 45.2%, HS 25%, p = 0.01). Patients with PH in functional class (FC; WHO criteria) III–IV had higher Vit D deficiency prevalence than those in FC I–II (86.7% vs 40.5%, *p* = 0.003). There was a significant linear correlation between the 6MWT and Vit D levels in PH (*p* < 0.01), but not in LVF (*p* = 0.69).

**Conclusions:**

Vit D levels were lower in patients with PH as compared to patients with LVF and HS and correlated directly with 6-min walk distance.

## Background

Vitamin D (Vit D), typically associated with phospho-calcium metabolism and bone disease, has been shown to influence several pathways that affect myocardial function and/or structure. As early as 1981, a reduction in cardiovascular mortality during the summer season was observed and it was attributed to higher Vit D levels produced by increased sun exposure [1].

Vit D receptors are present in several tissues associated with the cardiovascular system, such as cardiomyocytes, vascular smooth muscle and endothelial cells [1–3]. Vit D can suppress both the renin–angiotensin system (RAS) and inflammation [4, 5]. Vit D deficiency, and consequent elevated plasma renin activity, can induce ventricular remodelling and increase arterial blood pressure [6, 7]. In addition, Vit D locally reduces the expression of genes that increase myocardial hypertrophy [8]. Vit D may also prevent ventricular hypertrophy by increasing thrombomodulin and it has multiple effects on cardiomyocyte development and differentiation [9, 10].

Pulmonary hypertension (PH) is a low-prevalence, life-threatening disorder. Understanding PH physiopathology is essential to establish prognostic factors and to propose newer therapies. Vit D deficiency is more prevalent in patients with left ventricular failure (LVF) than in healthy subjects (HS) and it is strongly related to heart failure evolution [9]. The objective of this study is to compare Vit D levels in three groups: 1) patients with PH; 2) patients with LVF; and 3) a control population of HS. Also within the PH group we intended to correlate Vit D deficit with clinical variables with prognostic value such as the 6-min walk test (6MWT).

## Methods

### Study population

This cross-sectional study included three groups of patients: A) Patients with PH (consecutively recruited from the Pulmonary Hypertension program, Juan A. Fernández General Hospital, Buenos Aires, Argentina, followed from February 2011 to February 2016). The PH diagnosis was defined by heart catheterisation: presence of mean pulmonary arterial pressure (MPAP) ≥25 mmHg and pulmonary artery wedge pressure (PAWP) ≤15 mmHg; B) Patients with LVF (recruited from the Heart Failure program, Juan A. Fernández General Hospital, Buenos Aires, Argentina, from December 2011 to February 2016) defined by echocardiographic evidence of left ventricular ejection fraction (LVEF) < 35% and no right ventricular compromise; C) Healthy subjects (recruited among patients' relatives and hospital workers, from December 2011 to March 2016): no known medical conditions or chronic medications and normal physical exam, electrocardiogram and cardiac ultrasonography.

In the PH and the LVF groups, functional class (FC; WHO criteria), metres covered in the 6MWT and echocardiographic parameters (e.g., value of pulmonary artery systolic pressure, PASP, by the analysis of tricuspid regurgitation velocity; right ventricular systolic function, measured by tricuspid annular plane systolic excursion, [right ventricular dysfunction defined as TAPSE < 20 mm]; LVEF; among others) were analysed. In the PH group, plasma N terminal pro B type natriuretic peptide (NT-proBNP) level was analysed and a complete haemodynamic evaluation by right heart catheterisation was made.

In all groups, 8-h fasting blood samples were obtained in the morning. Serum Vit D levels were determined measuring 25-hydroxy Vit D using the electrochemoluminescence method (Cobas, Roche). Vit D deficiency was defined as a level of Vit D < 20 ng/ml. Patients being treated with Vit D, calcium supplements or receiving treatment for osteoporosis, patients with hyperparathyroidism or on dialysis and those who had been hospitalised for any reason in the previous 3 months, were excluded.

### Statistical analysis

Discrete variables were expressed as frequencies and percentages. Continuous variables were analysed with ANOVA and expressed as the mean with standard deviation. Association between qualitative variables was evaluated using the chi-square test with the Yates correction or the Fisher’s exact test. The correlation between continuous variables was analysed with the Spearman’s correlation coefficient. A *p* value < 0.05 was considered significant in all cases. Due to the exploratory nature of the study, *p*-values were not corrected for multiple testing.

## Results

Fifty three patients diagnosed with PH, 42 patients with LVF and 31 HS were included. General characteristics of patients with PH are shown in Table 1. A significantly higher haematocrit and lower basal haemoglobin saturation was found in the congenital PH group. Characteristics of the left ventricular failure and healthy subjects groups are shown in Table 2. Within the PH group, 48 patients corresponded to international classification Group 1 PH (16 idiopathic, 16 congenital, 9 associated with connective tissue diseases [CTD], 6 HIV and 1 drug/toxin-induced) and 5 to Group 4 (chronic thrombo-embolic pulmonary hypertension) [11]; only 36 patients (68%) of the whole PH group received specific treatment. Time from the onset of symptoms to diagnosis (right heart catheterization) was 41.1 ± 47 months, mean time from diagnosis to Vit D measurement was 86.3 ± 33 days; mean NT-proBNP at diagnosis was 610.2 ± 780 pg/ml. Sample distribution according to FC was: I 46.2%, II 25%, III 23.1% and IV 5.8%. Right heart catheterisation showed (mean ± SD): MPAP 50.6 ± 17 mmHg, right atrial pressure 8.6 ± 4 mmHg, PAWP 10.9 ± 3 mmHg, cardiac index 2.7 ± 1 l/min/m^2^, pulmonary vascular resistance 8.7 ± 4 Wood units (WU) and heart rate 81.9 ± 13/min. In the LVF group, mean LVEF was 34.3 ± 10% and mean left ventricular diastolic diameter was 65.6 ± 10 mm. In this group, 86.7% of patients were in FC I–II. Patients with PH showed a non-significant trend to higher and more advanced FC (FC III–IV in PH vs LVF; 28.8 vs 13.3%, *p* = 0.064).

**Table 1:** Basal characteristics of patients with PH divided by PH sub-group

PH subgroups		Total (*n* = 53)	Idiopathic (*n* = 16)	CTD (*n* = 9)	Congenital (*n* = 16)	HIV (*n* = 6)	Toxic (*n* = 1)	CTEPH (*n* = 5)	*p* value
Age (years; x ± SD)		40.8 ± 16.5	38.5 ± 19.1	53 ± 14.9	33.2 ± 15.5	42.7 ± 6.4	47	45.4 ± 11.7	0.10
Females (%)		73.6	81.25	100	75	66.6	0	40	0.04
FC (%)	I–II	71.2	62.5	44.4	100	33.3	100	100	0.01
	III–IV	28.8	37.5	66.6	0	66.7	0	0	0.01
Ht (%; x ± SD)		43.9 ± 8.9	42.8 ± 5.7	40.3 ± 5.3	50.7 ± 11.4 *	38.8 ± 9.1	42.1	42.1 ± 7.9	0.03
Urea (mg/ml; x ± SD)		34.49 ± 12.1	32.63 ± 9.6	32.50 ± 12.1	36.92 ± 17.1	33.33 ± 11.6	34	38 ± 5.2	0.91
Creatinine (mg/dl; x ± SD)		0.96 ± 0.9	0.76 ± 0.1	0.9 ± 0.1	1.29 ± 1.7	0.71 ± 0.1	1.4	0.98 ± 0.1	0.69
Sodium (mEq/Lt; x ± SD)		140.4 ± 2.6	139.7 ± 2.2	142.7 ± 2.2	140.4 ± 2.3	140.2 ± 2.8	141	138.8 ± 3.2	0.13
Bilirubine (mg/dl; x ± SD)		1.14 ± 1	1.14 ± 0.9	0.72 ± 0.1	1 ± 0.3	1.21 ± 1.3	1	2.08 ± 2.2	0.35
6-min walk test ( x ± SD)									
Distance (m)		392.5 ± 132.2	395.8 ± 114	337.2 ± 136.7	440.5 ± 114.5	294.1 ± 160.7	557	430 ± 146.2	0.11
Saturated O_2_, basal (%)		94.5 ± 5.3	96.7 ± 3.3	95.1 ± 1.2	90.8 ± 7.9 *	96.3 ± 1.3	98	95.25 ± 2.8	0.04
Desaturation		−6.20 ± 6	−5.33 ± 5.52	−8.33 ± 3.93	−9.07 ± 7.2	−2.67 ± 4.76	−1	−2.75 ± 4.99	0.14
Borg basal		0.42 ± 0.86	0.57 ± 1.01	0.86 ± 1.18	0.17 ± 0.57	0.42 ± 0.80	0	0	0.50
Borg final		3.35 ± 2.36	3.57 ± 2.34	3.93 ± 3.14	2.50 ± 2.06	4 ± 2.36	3	3 ± 2.58	0.79
Haemodynamic variables ( x ± SD)									
SBP (mmHg)		116.18 ± 17.76	110 ± 16.16	126.44 ± 18.37	114.45 ± 19.6	113.33 ± 17.51	135	117.5 ± 12.58	0.31
DBP (mmHg)		71.25 ± 10.59	71.23 ± 10.10	73.22 ± 15.03	67.73 ± 8.17	70 ± 8.94	85	75 ± 10	0.60
HR (bts/min)		81.9 ± 13	80.8 ± 15.1	83.4 ± 14.3	80 ± 11.4	88.2 ± 12.6	79	80.3 ± 13.5	0.89
MPAP (mmHg)		50.6 ± 17.7	55.7 ± 16.6	45.3 ± 6	56.7 ± 28.6	51.4 ± 7.7	29	38.8 ± 10.8	0.24
PAWP (mmHg)		10.9 ± 3.2	11 ± 2.7	9.8 ± 3.4	11.8 ± 4.7	12.2 ± 2.3	8	10 ± 2.5	0.63
RAP (mmHg)		8.6 ± 4.2	8.6 ± 4.5	8.1 ± 3.5	9.3 ± 3.2	10 ± 5.1	2	8.4 ± 5.7	0.66
PVR (Wood units)		8.71 ± 4.04	9.27 ± 2.34	8.88 ± 3.07	8.43 ± 7.05	10.44 ± 4.40	3.9	6.57 ± 3.48	0.56
CI (bts/min/m^2^)		2.73 ± 0.91	2.7 ± 0.69	2.12 ± 0.34	3.38 ± 1.33	2.4 ± 0.33	3.8	2.9 ± 1.13	0.07
Treatment									
Oral anticoagulation (*n*)		24	8	6	3	2	1	4	–
Calcium channel blockers (*n*)		11	4	3	2	1	0	1	–
PDE5 inhibitors (*n*)		34	11	6	10	4	0	3	–
Endothelin antagonists (*n*)		11	4	1	5	1	0	0	–
Prostanoids (*n*)		3	3	0	0	0	0	0	–

*p  < 0.05 vs rest of sub-groups. *BTS* beats; *CI* cardiac Index; *CTD* connective tissue disorder; *CTEPH* chronic thromboembolic pulmonary hypertension; *DBP* diastolic blood pressure; *FC* functional class; *Ht* hematocrit; *HR* heart rate; *MPAP* mean pulmonary artery pressure; *PAWP* pulmonary artery wedge pressure; *PDE* phosphodiesterase; *PVR* pulmonary vascular resistance; *RAP* right atrial pressure; *SBP* systolic blood pressure; *Vit D* vitamin D

**Table 2:** Characteristics of the left ventricular failure and healthy subjects groups

		PH (*n* = 53)	LVF (*n* = 42)	HS (*n* = 31)
Age (years)		40.8 ± 16.5	58.6 ± 10 *	40.5 ± 17
Females (%)		73.6	28.9 *	74.2
BMI (kg/m^2^)		26.2 ± 5	30.9 ± 6 *	25.2 ± 4
FC (%)	I–II	71.2	86.7	–
	III–IV	28.8	13.3	–
VitD (mg/dL)		19.25 ± 10.1 *	25.68 ± 12	28.8 ± 12
Ht (%)		43.9 ± 8.9	40.9 ± 7.5	40.6 ± 5.3
Urea (mg/mL)		34.49 ± 12.1	45.63 ± 16.3	33.6 ± 9.2 **
Creatinine (mg/%)		0.96 ± 0.9	1.04 ± 0.6	0.75 ± 0.3
Sodium (mEq/Lt)		140.4 ± 2.6	137.7 ± 6.2	140 ± 2.9
Total bilirubin (mg/dL)		1.14 ± 1	0.76 ± 0.8	0.57 ± 0.2 #
LVEF (%)		61.9 ± 7	34.3 ± 10 *	65.4 ± 8
LVDD (mm)		43.5 ± 6	65.6 ± 10 *	48.3 ± 23
LA (mm)		35.6 ± 8	46.84 ± 15 *	39.7 ± 7
SPAP (mmHg)		81.55 ± 3 *	43.91 ± 23	31.84 ± 15 **

*p < 0.05 vs. rest of groups; **p < 0.05 vs LVF; #*p* < 0.05 vs PH

**Fig. 1:**
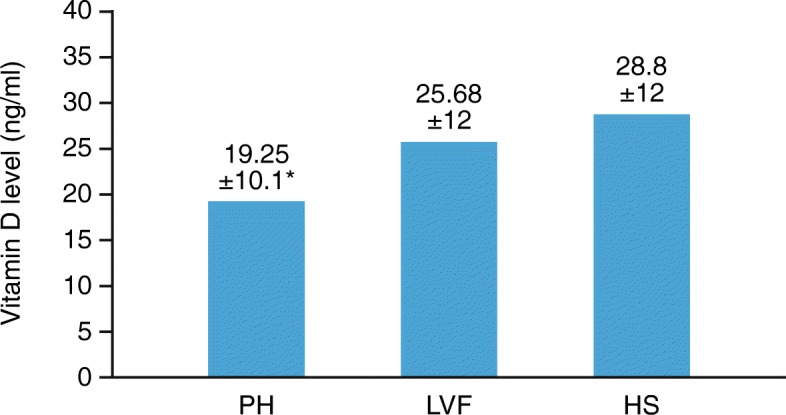
Vit D levels in the different study groups, expressed as ng/ml. *Significant differences were found between PH and LVF groups (*p* = 0.017) and between PH group and HS (*p* = 0.001). Difference between LVF and HS groups was not significant (*p* = 0.046). HS, healthy subjects; LVF, left ventricular failure; PH, pulmonary hypertension; Vit D, vitamin D

**Fig. 2:**
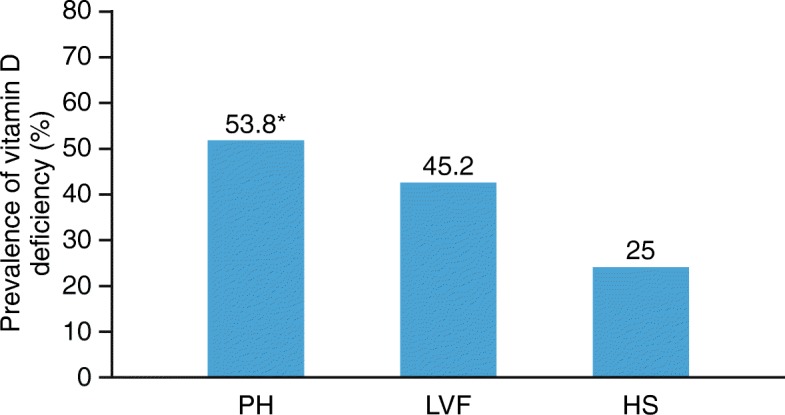
Prevalence of Vit D deficiency in the different study groups, expressed as percentages. *Differences were significant, *p* < 0.01, PH versus LVF and HS. HS, healthy subjects; LVF, left ventricular failure; PH, pulmonary hypertension; Vit D, vitamin D

Mean Vit D level was significantly lower in the PH group (19.25 ± 10 ng/ml) compared with the LVF group (25.68 ± 12 ng/ml, *p* = 0.017; Fig. 1) and HS (28.8 ± 12 ng/ml *p* = 0.001). There were no significant differences in Vit D levels between LVF and HS (HS vs LVF *p* = 0.46; Fig. 1) or among the different PH sub-groups (Table 1). The prevalence of Vit D deficiency was significantly higher in PH compared with LVF and HS (PH: 53.8% vs LVF: 45.2% and HS: 25%, *p* < 0.01; Fig. 2).

**Fig. 3:**
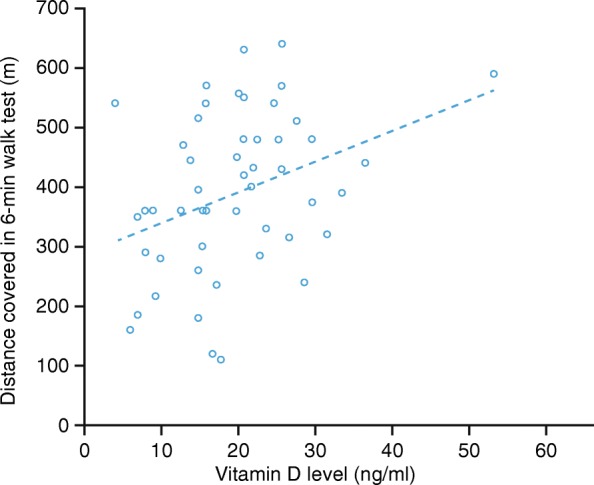
The 6MWT in the PH group. Significant correlation between distance covered in 6MWT (metres) and Vit D level (ng/ml), p < 0.01 (bilateral). Spearman’s rho correlation coefficient 0.36. 6MWT, 6-min walk test; PH, pulmonary hypertension; Vit D, vitamin D

In the PH group, patients in FC III–IV showed greater prevalence of Vit D deficiency compared with patients in FC I–II (86.7% vs 40.5%, *p* = 0.003). In this same group, our study found a tendency to a greater prevalence of Vit D deficiency in patients with right ventricular dysfunction (*p* = 0.052). In addition, a higher prevalence of Vit D deficit was found in patients with high PASP measured by echocardiography (*p* = 0.038). A significant linear correlation (r = 0.36; *p* = 0.01) was found between metres covered in the 6MWT and Vit D levels in the PH group, but not in the LVF (*p* = 0.69; Fig. 3). No significant differences were found between PH group and LVF group in the distance covered in 6MWT (392.5 ± 132 m, PH vs 409.1 ± 99 m, LVF, *p* = 0.5).

## Discussion

Pulmonary hypertension is a disabling and potentially fatal disorder characterised by a progressive increase in pulmonary vascular resistance leading to right ventricular impairment and heart failure [11]. Main treatments act on known physiopathological pathways: endothelin, nitric oxide, prostacyclin and the coagulation system. Patients may also be affected by other complications, such as Vit D deficiency [12, 13].

Vit D appears to be related to arterial blood pressure control through different pathways and is inversely correlated with serum renin activity [14]. Evidence suggests a link between Vit D and cardiovascular disease, including experimental studies that identify Vit D receptors in vascular smooth muscle cells, endothelial cells and in cardiac muscle tissue [15]. Although not clearly related, some previous studies suggest a higher prevalence of Vit D deficiency in patients with PH [13, 16–18].

Our study shows that mean Vit D levels are significantly lower in patients with PH compared with both HS and patients with LVF. In addition, prevalence of Vit D deficiency is significantly higher in patients with PH compared to HS and to patients with LVF. Vit D deficiency in the PH group was associated with poorer FC and worse echocardiographic parameters. No correlation between Vit D levels and haemodynamic parameters measured by right heart catheterisation was found; this may be because diagnostic catheterisation differed temporarily with Vit D dosage. Significant correlation between exercise capacity measured with the 6-min walk distance test and Vit D levels was observed, establishing that the lower the Vit D levels, the shorter the distance covered. The 6MWT is a well-known prognostic factor in patients with PH.

Interestingly, a significant difference between Vit D levels in PH and LVF groups was found. Although an activation of the RAS and the sympathetic nervous system may be found in both LVF and PH, no studies have described differences in the magnitude of these phenomena between both disorders [20]. Vit D deficiency and its relationship with these pathways might be different in both diseases. Although Vit D receptors in myocardial cells and cardiac fibroblasts exist, it remains unknown if their function or concentrations are different between left and right ventricle [21, 22].

With some exceptions, the characteristics of the PH patient cohort were similar to those found in international registries [23]. They differed in age and FC at the time of diagnosis: both came out to be somewhat lower in our study than in most available data. Vit D levels and demographic characteristics in our LVF population are similar to those found in other studies [24–25]. Vit D deficiency is more frequently observed in elderly patients. Despite being younger than patients in the LVF group, patients with PH had lower Vit D levels [26]. Only 68% of patients included in our study received PAH-specific treatment; unfortunately, due to bureaucratic considerations in our country, there is a considerable delay between the PAH diagnosis and the actual access to specific treatment drugs.

As with other chronic cardiovascular conditions, we do not know if Vit D deficit is a cause or a consequence of PH. There is an established relationship between inflammatory factors and Vit D, with Vit D being protective in inflammatory conditions [27–29].

Some limitations of our study should be addressed. Catheterization and hemodynamic analysis differed temporarily with Vit D dosage, a fact that could represent a potential source of bias. Also, the reduced sample size and did not allow us to correlate specific PAH treatment with Vit D levels or to adequately compare Vit D levels between different PH subgroups.

## Conclusions

At present, there is limited scientific evidence of the relationship between PH and Vit D deficiency. This study shows that patients with PH have lower Vit D levels and a higher prevalence of Vit D deficiency compared to HS and to patients with LVF. In addition, a relationship between Vit D deficiency in patients with PH and poor prognosis-associated variables was found. It will be interesting to establish whether patients with PH and Vit D deficiency should be treated with Vit D supplements to improve disease prognosis.

## Data Availability

The datasets used and/or analysed during the current study were complete (no missing values) and are available from the corresponding author on reasonable request.

## Abbreviations

6MWT: 6-min walk test
CTD: Connective tissue diseases
FC: Functional class
HS: Healthy subjects
LVEF: Left ventricular ejection fraction
LVF: Left ventricular failure
MPAP: Mean pulmonary arterial pressure
PAWP: Pulmonary artery wedge pressure
PH: Pulmonary hypertension
RAS: Renin-–angiotensin system
TAPSE: Tricuspid annular plane systolic excursion
Vit D: Vitamin D
